# Ewing Sarcoma in Nepal Treated With Combined Chemotherapy and Definitive Radiotherapy

**DOI:** 10.1200/JGO.19.00015

**Published:** 2019-03-27

**Authors:** Anjani Kumar Jha, Pradeep Neupane, Manohar Pradhan, Krishna Sagar Sharma, Sadina Shrestha, Padma Raj Sigdel, Sigbjørn Smeland, Øyvind S. Bruland

**Affiliations:** ^1^ BP Koirala Memorial Cancer Hospital, Bharatpur, Nepal; ^2^ Norwegian Radium Hospital, Oslo University Hospital, Oslo, Norway; ^3^ Institute of Clinical Medicine, University of Oslo, Oslo, Norway

## Abstract

**PURPOSE:**

To our knowledge, we conducted the first prospective oncologic clinical trial in Nepal aimed at providing state-of-the-art chemotherapy to patients with Ewing sarcoma. The efficacy of external-beam radiotherapy (RT) as the sole local treatment modality was explored and deemed justified as a result of the lack of available advanced tumor-orthopedic services in Nepal.

**PATIENTS AND METHODS:**

Twenty patients, 11 female and 9 male patients between the ages of 6 and 37 years, with newly diagnosed Ewing sarcoma were enrolled. Neoadjuvant combination chemotherapy, comprising well-established drug combinations, was administered in five courses before external-beam RT, during which one course of etoposide and ifosfamide was given. After RT, six additional chemotherapy courses were scheduled.

**RESULTS:**

RT was tolerated well, providing rapid symptom relief and local tumor control, with no pathologic fractures observed among the 15 patients who received such treatment. Eleven patients completed the entire treatment protocol; seven patients were under continued follow-up, with no evidence of disease in six patients at a median follow-up time of 2.3 years (range, 1.3 to 3.1 years) and one patient alive but with a regional recurrence. Four patients experienced metastatic relapse and died as a result of their disease. Three treatment-related deaths linked to toxicity from chemotherapy occurred. Four of the six patients who refused to complete the treatment protocol and were lost to follow-up experienced progressive disease and were assumed dead.

**CONCLUSION:**

This study was feasible with RT as the sole local treatment modality in combination with chemotherapy. As a result of the high number of patients lost to follow-up, no firm conclusions can be drawn, but the majority of the patients who completed treatment obtained durable long-term remissions.

## INTRODUCTION

Ewing sarcoma (ES) belongs to a rare family of tumors composed of small round cell tumors (SRCTs) arising primarily in the bone and most frequently affecting children, adolescents, and young adults.^1-3^ Epidemiologic studies have shown a lower incidence of ES in populations of African and East Asian origins compared with white populations.^4^ Information on the clinical characteristics of patients with ES in the developing world is sparse. Two epidemiologic studies are reported from Iran and India.^5,6^ To our knowledge, three studies, all with a retrospective design, have reported on clinical outcomes after multimodality treatment.^7-9^

**CONTEXT**

**Key Objective** Experiences and outcomes are reported from, to our knowledge, the first prospective oncologic clinical trial to be conducted in Nepal providing state-of-the-art chemotherapy to patients with Ewing sarcoma in combination with external-beam radiotherapy as the sole local treatment modality.
**Knowledge Generated** Twenty patients were enrolled, including four patients with primary metastatic disease. Three treatment-related deaths were encountered during chemotherapy. Eleven patients completed the entire scheduled combined treatment. Six of these patients have experienced neither local relapse nor metastasis, with a median follow-up of 2.3 years.
**Relevance** As a result of six patients who were lost to follow-up before having completed chemoradiotherapy, no firm conclusions can be made.

In Europe and North America, survival has substantially improved over the past couple of decades, with an expected 5-year overall survival rate of 65% to 75% for patients with localized disease at presentation and 30% for patients with primary metastatic ES.^10,11^ Multiagent chemotherapeutic regimens effectively eradicate systemic micrometastases in ES and most often induce a rapid response on the primary tumor site, with shrinkage of the soft tissue components.^12,13^ A randomized study comparing the vincristine, doxorubicin, cyclophosphamide, and actinomycin C regimen with the vincristine, doxorubicin, cyclophosphamide, and actinomycin C regimen plus ifosfamide and etoposide demonstrated that, in nonmetastatic patients, the six-drug regimen offered the best survival probability.^14^ The outcome for Scandinavian patients in the Italian Sarcoma Group (ISG)/Scandinavian Sarcoma Group (SSG) III protocol^15^ represents a substantial improvement compared with the previous trials SSG IV^16^ and SSG IX,^17^ comparing favorably with the results from other intergroups.^14,18,19^ In patients with limited metastatic disease at presentation, a more aggressive approach that included myeloablative treatment showed survival benefit.^20^

The current Nepali-Norwegian Ewing Sarcoma Study (NEWS) initiative was launched to explore the feasibility of implementing a prospective clinical study protocol providing multidisciplinary treatment to Nepalese citizens with ES and to report its outcomes. The study aimed to facilitate the referral of these patients to the BP Koirala Memorial Cancer Hospital (BPK) and to provide an evidence-based combination chemotherapy regimen administered at BPK. Another important goal was to gain systematic experience on the effectiveness of definitive external-beam radiotherapy (RT) as the sole local treatment modality to compensate for the lack of state-of-the-art limb-sparing tumor-orthopedic surgical service in Nepal. NEWS represents the collaboration between BPK and the Norwegian Radium Hospital, Oslo University Hospital, Norway. To our knowledge, this was the first prospective oncologic clinical study conducted in Nepal. The combination chemotherapy was adapted to the BPK experiences and resources, combining five effective drugs for ES that were successfully applied in the ISG/SSG III trial.^15^

Advances in both surgery and RT have resulted in better local control, fewer adverse effects, and the avoidance of amputation in the majority of patients.^1,10^ Patients with ES in the axial skeleton have a worse prognosis compared with those with tumors in the extremities.^21^ Surgical treatment of axial ES is often associated with considerable loss of function and difficulty in obtaining wide margins.^22-24^ Thus, postoperative RT is widely used when the histologic response to preoperative chemotherapy is unsatisfactory^25^ and/or surgical margins are regarded as inadequate.^1^ More recently, particle-beam RT with protons has been reported to yield favorable local control rates as single treatment of the primary tumor.^26^ The risk of a pathologic fracture after RT, when the primary tumor is localized in a weight-bearing bone, is an unsettled concern. Even the most sophisticated RT may have serious acute and late complications, including secondary malignancies,^27,28^ which are of particular concern among children with ES. Evidence regarding strategies for optimal local disease control is still somewhat contradictory; some institutions insist on attempting free-margin surgical resection whenever possible, whereas others propose definitive RT as an equally effective alternative.^23,29^ This article reports the first outcomes of the NEWS protocol related to patient compliance during treatment and follow-up; practical and logistical aspects; and the impacts of intensive polyagent chemotherapy and definitive RT on local tumor control, metastases-free survival, and adverse events.

## PATIENTS AND METHODS

### Patients

**FIG 1:**
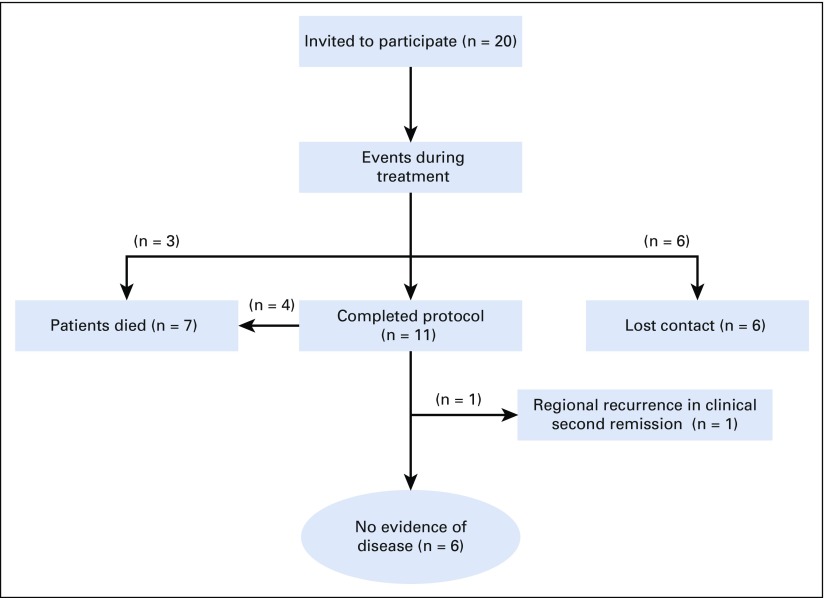
Overview of patients enrolled in the trial.

Over a 2-year period, in total, 20 patients were invited to participate (Fig 1) and informed about the nature of the disease, as well as the treatment’s expected benefit and adverse effects. All 20 patients (11 females and nine males) accepted and signed the written informed consent form. The enrollment was from August 2014 to October 2016. Table 1 lists the patient age (at diagnosis) and sex distribution, the anatomic site of primary tumor with largest diameter, and the evidence of primary metastases.

**TABLE 1:**
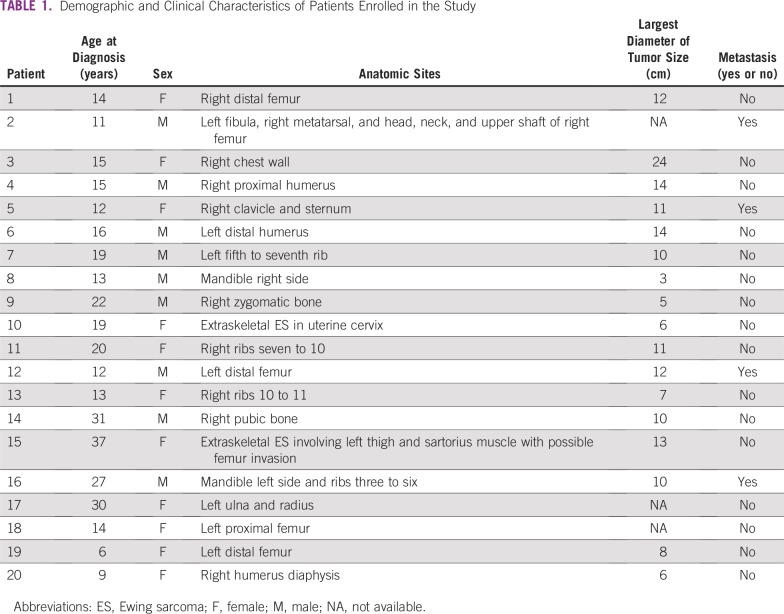
Demographic and Clinical Characteristics of Patients Enrolled in the Study

The inclusion criteria included histologically proven SRCT compatible with ES, age between 5 and 60 years, normal hepatic and renal functions, WBC count of 3.0 × 10^9^/L or greater, and platelet count of 100 × 10^9^/L or greater. Chemotherapy had to be started within 4 weeks of a conclusive histologic diagnosis. Chest x-ray and computed tomography images were mandatory, and in some cases, magnetic resonance imaging (MRI) of the primary tumor was used to estimate the largest tumor length.

The exclusion criteria included previous malignancy other than basal cell carcinoma of the skin or noninvasive carcinoma of the cervix, medical contraindications to the cytostatic agents and dose levels in question, major psychological or psychiatric disorders, and a priori expected lack of compliance to treatment and scheduled follow-ups. Patients with synchronous metastatic diseases were eligible if it was possible to include lesions in separate RT target volumes (see later discussion).

Case report forms were developed on demographic and clinical variables at inclusion to document completion of chemotherapy cycles, capture RT details and adverse events, and document the clinical status on scheduled follow-up visits (Data Supplement). The clinical outcomes were classified as follows: no evidence of disease (NED), progressive disease (distant relapse or local recurrence), died of disease, or lost to follow-up (LFU).

### Pathologic Diagnosis

Pathologic diagnosis was based on an open surgical biopsy or core-needle biopsies. Tissue was fixed in 10% buffered formalin for microscopic evaluation. Two experienced pathologists confirmed the diagnosis. Monoclonal antibodies were used for immunohistochemistry (IHC) analyses that comprised testing for expression of CD99 in all except one patient (Table 2). However, a standardized ES panel was not systematically used. Table 2 lists the IHC details of the 20 patients; IHC was performed at BPK in eight patients, with the remaining 12 patients having IHC performed at a private laboratory in India.

**TABLE 2:**
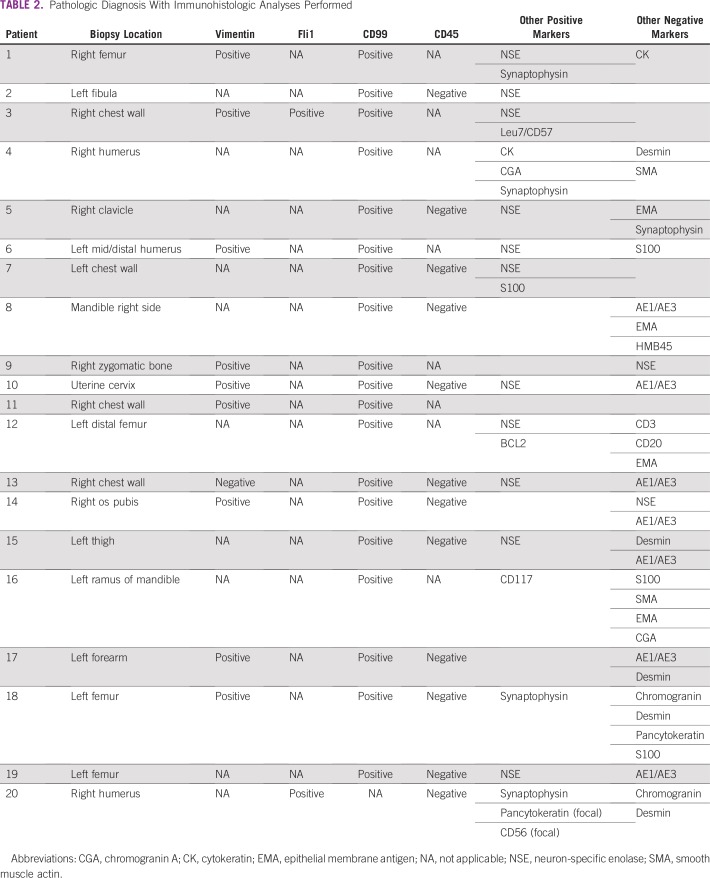
Pathologic Diagnosis With Immunohistologic Analyses Performed

In 12 patients, digital images from hematoxylin and eosin–stained and IHC slides were anonymously e-mailed to the Norwegian Radium Hospital for review to confirm a morphologic diagnosis and an IHC profile compatible with ES/SRCT. Molecular pathology, with the ES diagnostic translocation marker, was not applied in this study.

### Chemotherapy

**FIG 2:**
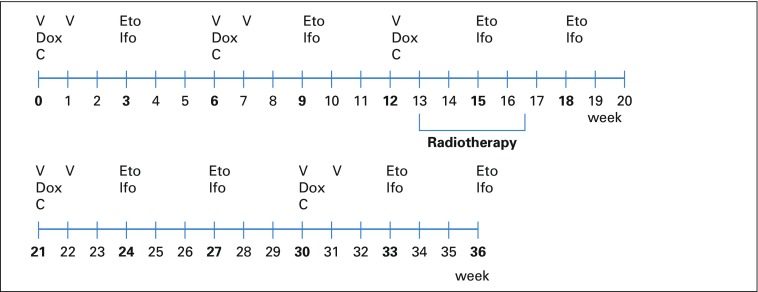
Outline of the Nepali-Norwegian Ewing Sarcoma Study treatment protocol. C, cyclophosphamide (1,200 mg/m^2^ as a 30-minute intravenous [IV] infusion); Dox, doxorubicin (40 mg/m^2^/d as a 4-hour IV infusion on days 1 and 2; total dose, 80 mg/m^2^ in 2 days; total cumulative dose, 400 mg/m^2^); Eto, etoposide (150 mg/m^2^/d as 2-hour IV infusion; total dose, 450 mg/m^2^ in 3 days); Ifo, Ifosfamide (3,000 mg/m^2^ over 21 to 24 hours as 3-day continuous IV infusion; total dose, 9,000 mg/m^2^ in 3 days); V, vincristine (1.5 mg/m^2^ IV push; maximum, 2 mg).

Figure 2 presents the chemotherapy combinations with scheduling of the different combinations, as well as individual drug dosages based on the experiences from the ISG/SSG III trial cycles^15^ that combined vincristine, doxorubicin, cyclophosphamide, ifosfamide, and etoposide. Dactinomycin was excluded from the current chemotherapy regimen as a result of its significant addition to radiation toxicity and the sparse documentation on its antitumor effect on ES. A course of etoposide and ifosfamide chemotherapy was administered during the RT course. In total, 13 cycles of chemotherapy were planned over 36 weeks (Fig 2).

### RT

An aim of NEWS was to explore the feasibility and effectiveness of RT as the sole local treatment modality with prospective registration of tumor control and normal tissue complications. The fractionation schedule was 1.5 Gy, given twice daily with a 6-hour minimum between fractions, for a total dose of 54 Gy interpolated between chemotherapy cycles^15^ (Fig 2). Because of the potential risk of pathologic fracture among patients whose primary tumor was located in weight-bearing bones, patients were instructed to avoid weight-bearing stress and to use crutches. A backup strategy, involving an orthopedic intervention in the event of a pathologic fracture, was in place at BPK.

RT was documented in a separate case report form (Data Supplement). Acute toxicity and long-term sequelae of RT were documented. The radiation toxicity scoring was done according to the Radiation Therapy Oncology Group/European Organisation for Research and Treatment of Cancer Radiation Morbidity Scoring Schemes. Doses administered to critical organs were calculated based on the relevance of the actual RT administered, which should not exceed the following maximum values: spinal cord, 45 Gy; heart, 30 Gy to more than 50% of its volume; liver, 30 Gy to more than 50% of its volume; lung, 20 Gy to the whole lung; and kidney, 14 Gy to the whole kidney.

RT was administered with high-energy 6-MV photons from a Varian 600 CD Linear Accelerator (Varian Medical Systems, Palo Alto, CA) with individual-dose planning to optimize treatment (eg, multiple beams with secondary-field shaping, individual filters, wedges). Computerizing the planning, using a map of isodose distribution on at least three slices, was required, including one central (reference plane) slice and two slices between the central and the peripheral planes. Whenever possible, some portion of the circumference of an extremity was excluded from the treatment volume to reduce the risk of distal or peripheral edema. A shrinking-field technique was applied, with target volume I encompassing the original tumor volume (gross tumor volume) with a 2-cm margin (clinical target volume) that received a dose of 42 Gy in 28 fractions. If a substantial shrinkage of the primary tumor during the first chemotherapy courses was observed, the initial soft tissue extensions seen at diagnosis were not part of the delineated boosted target volume II (eg, when the soft tissue mass expanded into thoracic or abdominal cavities). However, the entire bone or bone marrow extension at primary diagnosis was always used to guide the delineation of target volumes. An additional 12-Gy dose was given to target volume II, comprising the clinically and radiographically evident tumor at the start of RT (after neoadjuvant chemotherapy) with a 2-cm margin. A total accumulated dose of 54 Gy in 36 fractions was given to the gross tumor volume.

Four patients (patients 2, 5, 12, and 16) had synchronous metastases. Three patients with skeletal lesions (suspected as metastasis but not biopsied) were administered RT as separate target volumes encompassing these lesions, similar to those for the expected primary tumor. Patient 12 had bilateral lung metastases and received 12 doses of 1.5 Gy to both lungs.

### Follow-Up Visits

Visits were scheduled at 3, 6, and 9 months after completion of the chemotherapy cycle, with subsequent planned consultations every 6 months for up to 5 years of follow-up. Clinical examinations of the primary tumor site and the regional lymph nodes, as well as radiation toxicity scoring, were mandatory. In addition, x-rays of the affected bone or primary tumor site, conventional blood tests, and chest x-rays were planned. Computed tomography (or MRI if available) was performed in patients with suspected local relapse or metastases.

### Ethical Aspects

A formal protocol was written for this prospective study that was presented to and approved by the administration of BPK. The Ethical Review Board of the Nepal Health Research Council approved this study on August 14, 2014 (Registration No. 126/2014).

## RESULTS

### Patient Demographic and Disease-Related Information

The median age at diagnosis was 15 years (range, 6 to 37 years), with a 1.2 female predominance. The anatomic sites of the primary tumors were femur (n = 4), pubic bone (n = 1), fibula (n = 1), thoracic wall or costae (n = 4), clavicle (n = 1), craniofascial bone (n = 3), humerus (n = 3), and forearm (n = 1). Patient 15 may have had a large extraskeletal ES in the musculus sartorius, albeit with an invasion into the adjacent femur. Patient 10 had a primary tumor in her uterine cervix (ie, anticipated to be soft tissue ES; Table 1). However, in this patient, the panel of IHC analyses did not contain muscle markers (Table 2). Hence, a rhabdomyosarcoma diagnosis could not be ruled out. Table 1 also lists the available information on the largest tumor diameter for 17 patients, varying between 3 cm to 24 cm, but with no information for three patients.

Table 2 lists the results of the IHC. All patients had tumor morphology with classic SRCTs on conventional hematoxylin and eosin staining. Unfortunately, the completeness of the tested IHC panel varied substantially. Hence, a definite ES diagnosis (eg, with the sound exclusion of rhabdomyosarcoma or primary bone lymphoma) could not be determined in eight patients.

Patient 2 had his anticipated primary tumor in the left fibula and was diagnosed with synchronous, metastatic disease comprising two tumorous foci located in the contralateral extremity (foot and proximal femur). Patient 16 had a 10-cm primary tumor in the left mandibular ramus and an additional lesion suspected to be a metastasis involving costae 3 to 6. Both anatomic sites were administered RT as separate clinical target volumes. Patient 5 had an 11-cm tumor in her right clavicle and presented with possible metastases in both the manubrium of the sternum and a small lesion in the right parietal skull, diagnosed by MRI.

### Compliance With Protocol and Clinical Status During Follow-Up

Figure 1 and the Data Supplement provide details on the enrollment, completeness of chemotherapy and RT according to the protocol, events during treatment, and available information on the clinical status during follow-up. All four patients enrolled with primary metastatic disease have died of disease.

Eleven patients completed the entire NEWS protocol as scheduled, without substantial delays or deviations regarding chemotherapy doses and RT (Figs 1 and 2). Four of these patients (patients 5, 12, 15, and 20) subsequently experienced metastases and died of disease. Patient 5 developed progression in the known metastatic sites of the manubrium of the sternum and the skull. Patient 12 experienced progression of his primary metastatic disease in both lungs. Patients 15 and 20 died as a result of disseminated metastases to both lungs diagnosed 12 and 3 months, respectively, after completion of NEWS.

One of the 11 patients (patient 19) who completed the entire NEWS protocol and had NED at the end of the therapy experienced a regional recurrence in her left inguinal region 6 months later. This was biopsied and showed SRCT morphology that this time tested positive for desmin expression. After response to second-line palliative chemotherapy, she remained symptom free at another 6-month follow-up.

The remaining six patients who had thus far not experienced a relapse all belonged to the subcohort of 11 patients who had complied with the entire NEWS protocol. The clinical data were updated in January 2018 (Fig 1 and Data Supplement). The median follow-up time was 2.3 years (range, 1.3 to 3.1 years) for the six patients with NED who were not LFU.

Among the six LFU patients, four had documented disease progression and were assumed to have died of disease. Patient 6 experienced progressive disease during chemotherapy and was found to be ineligible for RT as a result of massive lymphedema of the affected upper extremity. He was LFU after cycle 11 of chemotherapy. Five patients refused to comply with and complete the treatment and were LFU at various treatment stages. Despite various and repeated efforts to contact the patients and/or their families, no information on the state of their disease could be gathered.

### Chemotherapy Compliance and Toxicity

The Data Supplement provides the number of completed chemotherapy cycles, information on substantial delays, and available information on serious adverse events. During the scheduled chemotherapy courses (Fig 2), three patients (patients 2, 3, and 16) experienced severe hematologic toxicity and died in a state of assumed febrile neutropenia (not at the treating hospital; Fig 1). Dose reductions in the 10% to 20% range were deemed necessary in three patients (Data Supplement).

### RT Experience

As shown in the Data Supplement, 15 of 20 patients received the scheduled RT, including the boost-volume RT, for the specified total accumulated 54-Gy dose. Patient 2 also received the same fractionated RT to the two assumed metastatic disease sites (right proximal femur and right foot).

Systematic information on primary tumor control and normal tissue complications is far from complete because of the high number of patients LFU. However, among the patients who completed the entire treatment protocol, all had rapid and significant pain relief and considerable tumor shrinkage during the RT course. None of these patients experienced local tumor progression, and at the scheduled follow-up visits, no pathologic fractures were diagnosed at irradiated primary tumor sites.

No functional disability was observed, but most of the patients treated for ES in the lower limbs had mild peripheral distal edema, although it was of short duration. This observation is interesting because several patients had larger tumors, which posed considerable challenges in sparing a sufficient part of the circumference of the treated extremity from the full irradiation dose.

## DISCUSSION

ES is an RT-sensitive tumor,^11,23,24,30^ but surgery is considered the standard of care for local control.^21,29,31^ However, for inoperable tumors and tumors not operated with sufficient margins, RT adds substantial benefits to local control. In addition, RT techniques are rapidly evolving, improving the therapeutic index. To our knowledge, among patients with ES in the axial skeleton, no studies have proven a convincing benefit of surgery compared with RT regarding local control.

When planning this study, advanced tumor-orthopedic surgery involving reconstructions with internal prostheses was not an available option in Nepal. Currently, only one hospital in Kathmandu offers such operations. However, the patient has to cover all expenses. Thus, this study’s exploratory hypothesis was that, in ES, external-beam RT would be as equally effective as surgery in local tumor control and overall survival when administered sequentially with an effective multidrug chemotherapy regimen. Given this setting, the clinical trial was deemed ethically acceptable. No a priori sample size calculations were made because no previous outcome data on ES outcome in Nepal were available.

An earlier timing of RT, provided sequentially with chemotherapy, and the introduction of hyperfractionated and accelerated RT as 1.5 Gy administered twice daily^15^ have composed the advocated strategy in the SSG and ISG. However, the true benefit of twice-daily RT has not been adequately studied, and most centers currently administer one fraction of 1.8 to 2.0 Gy/d.

The NEWS treatment protocol was feasible, and the oncologic outcomes were satisfactory for the 11 patients who completed the protocol, with no or minor dose reductions and without significant delays. Among these patients, six (55%) still had NED at a median follow-up of 2.3 years from the start of chemotherapy. Four of the 20 enrolled patients had primary metastatic disease, some of the primary tumors were of considerable size, and nine patients had axial tumors. These are all adverse prognostic factors for outcome.

All eligible patients seen at BPK during the actual period of accrual were invited to participate independent of economic capability. However, we cannot rule out a selection bias for patients who could afford flying to India and paying for their entire treatment costs.

Despite the limited number of patients in this study, the fact that neither local disease progression nor any pathologic fracture was diagnosed is an important observation. This may also convey relevant information for sarcoma centers currently considering advanced surgical treatment of axial ES with significant functional disabilities as the best therapeutic option.

The NEWS study has several limitations. The high percentage of patients LFU (six of 20 patients) allows no sound conclusions on outcome to be drawn. As expected, given the socioeconomic situation in Nepal, tracing individual patients during follow-up turned out to be a major challenge, despite considerable efforts to re-establish contact by phone and postal mail.

The key message of this article is that it was feasible to conduct the ambitious clinical NEWS study at the BPK, with several patients still in first complete remission. This leads to the conclusion that international collaboration, as the advocated current approach in the Western world,^11^ might facilitate future collaborations in other cancer diagnoses for the benefit of both patients and oncologists in Nepal.

## Appendix

### Financial Aspects and External Funding

External funding was provided by the Norwegian Radium Hospital Research Foundation to reimburse the patients without financial resources for their chemotherapy drug expenses.BP Koirala Memorial Cancer Hospital covered all other expenses for radiologic examinations, biopsies, administration of chemotherapy with hospital costs, management of adverse events, radiotherapy, and the planned outpatient follow-up visits. None of the 20 patients were able to raise sufficient money to pay for chemotherapy.
